# Skin and systemic infections in children with atopic dermatitis: review of the current evidence

**DOI:** 10.3389/fped.2025.1513969

**Published:** 2025-05-14

**Authors:** Rodrigo Lomelí-Valdez, Luz Orozco-Covarrubias, Marimar Sáez-de-Ocariz

**Affiliations:** Dermatology Department, National Institute of Pediatrics, Mexico City, Mexico

**Keywords:** atopic dermatitis, skin infection, staphylococcal skin infection, viral skin infection, bacterial skin infection, fungal skin infection

## Abstract

Atopic dermatitis is a chronic, pruritic inflammatory skin disorder that affects approximately 2%–42% of children worldwide. Its course is frequently complicated by secondary bacterial, viral, and fungal infections, which can exacerbate disease severity and hinder treatment outcomes. These infections are thought to arise due to a disrupted skin barrier, reduced antimicrobial peptide production, alterations in the skin microbiome, and Th2-dominant inflammatory response. Identifying the most prevalent and pathogenic microorganisms in patients with AD is critical for early diagnosis, effective management, and prevention of complications. This review provides an updated synthesis of current knowledge on the infectious agents implicated in AD pathogenesis, summarizing recent findings on the epidemiology, microbial interactions, and immune mechanisms involved. Furthermore, it provides an overview of the latest therapeutic strategies for managing AD and its associated infections. By integrating recent insights into pathogenesis and treatment, this study offers a comprehensive perspective on the evolving landscape of AD management in children.

## 1 Introduction

Atopic dermatitis (AD) is the most common inflammatory skin disease in children, with a prevalence ranging from to 2% to 42%, depending on country-specific reports (1–3). Its pathogenesis is complex and multifactorial, involving both endogenous and exogenous factors. Children with AD are more susceptible to certain types of infections, including bacterial, viral, and fungal infections, which initially affect the skin, but may spread systemically if not properly managed (4).

### 1.1 Pathogenesis

**Figure 1:**
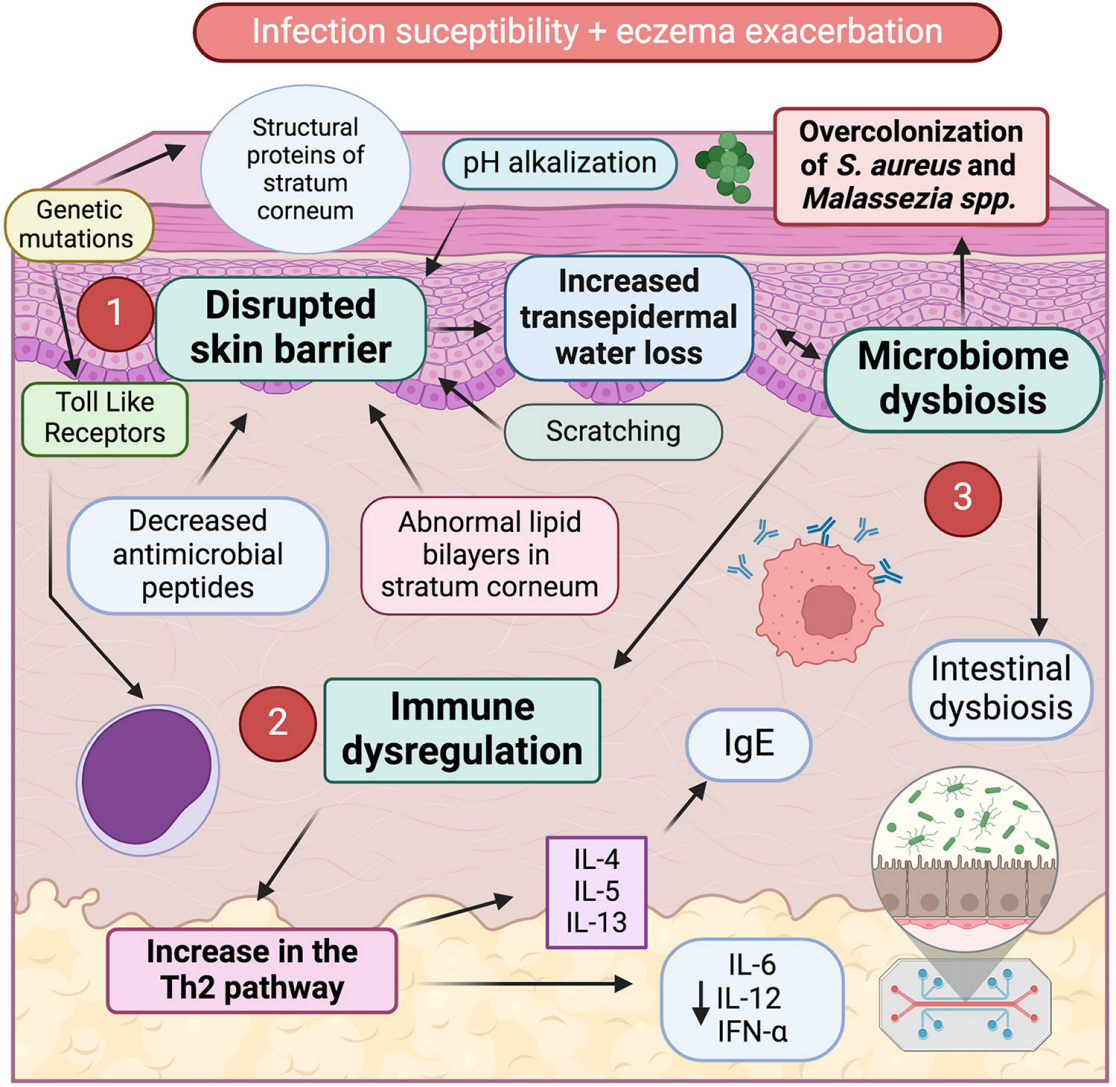
Susceptibility to infections in AD is driven by three main factors: disrupted skin barrier, immune dysregulation and microbiome dysbiosis. 1. Disrupted skin barrier is characterized by increased transepidermal water loss due to a) genetic mutations in structural proteins of stratum corneum (e.g., filaggrin, loricrin, claudin); b) pH alkalinization; c) abnormal lipid bilayers in the stratum corneum; d) reduced antimicrobial peptide production; and e) mechanical damage from scratching. 2. Immune dysregulation, marked by a Th2 predominant immune response with elevated production of interleukins linked to pruritus and heightened IgE synthesis. 3. Microbiome dysbiosis, characterized by overcolonization of *S. aureus* and *Malassezia spp.* in the skin surface, and intestinal dysbiosis, which might contribute to both exacerbate inflammation and facilitate infections in AD patients.

The prevalence of skin infections is higher in children with AD compared to healthy individuals (5). Several mechanisms contribute to this increased susceptibility: increased transepidermal water loss, altered pH of the skin, disrupted lipid distribution, immune dysregulation, microbiome dysbiosis, and scratching (4) (Figure 1).

#### 1.1.1 Skin barrier defects

In AD, the skin barrier is primarily altered in the stratum corneum, leading to dysfunction and dryness, allowing easier penetration of irritants, microorganisms, and other antigens (6). This dysfunction involves increased transepidermal water loss and disruptions in molecules such as filaggrin, loricrin, ceramides, fatty acids, cholesterol, involucrin, and claudins (1). These molecules are essential for maintaining the cohesion of the stratum corneum, its hydration, an acidic skin surface pH, and preventing overcolonization by pathogenic bacteria, such as *Staphylococcus aureus* (7).

Lipid lamellar bilayers of stratum corneum of children with AD differ from those in healthy children. There is a reduction in essential lipids, such as sphingolipids and free fatty acids. This can lead to larger intercellular spaces, disruption of the permeability barrier and increased transepidermal water loss. Decreased degradation of filaggrin leads to reduced levels of some components of the natural moisturizing factor (NMF), like pyrrolidone carboxylic acid and urocanic acid, which contribute to stratum corneum acidification (8). The cornified envelope is composed of various molecules: loricrin, filaggrin, and involucrin, which are crosslinked by transglutaminase with K1, K10, and desmosomal proteins such as envoplakin and periplakin (9). Genetic mutations in these molecules can lead to barrier dysfunction, with the filaggrin gene (OMIM *135940, FLG) being the most frequently altered, affecting up to 30% of patients with AD (10).

#### 1.1.2 Immune dysregulation

Keratinocytes in patients with AD secrete higher levels of IL-25 and IL-33, which induce a Th2 response with the secretion of IL-4, IL-5, and IL-13 (4). This Th2 dominance, along with decreased IL-17, lowers antimicrobial peptide production, predisposing patients to skin infections (11). This decrease in antimicrobial peptide production has also been observed in patients with similar skin barrier defects such as ichthyosis (12). However, psoriasis, another inflammatory dermatosis linked to a defective skin barrier, does not show higher infection rates compared to patients with AD (13). This might be explained because antimicrobial peptides are increased in the skin with psoriasis (14).

Total IgE levels are elevated in approximately 80% of AD cases (15). In the literature, AD associated with elevated IgE is referred to as extrinsic AD, while cases with normal IgE levels are classified as intrinsic (16). The increase in IgE is directly linked to a heightened Th2 response, driven by increased antigen presentation through an impaired skin barrier. The severity of AD correlates with IgE levels (17).

Severe AD-associated infections have been linked to toll-like receptor 2 (TLR-2) polymorphisms, which increase susceptibility to skin infections by reducing IL-6 and IL-12, and T-cell immunity (18). Additionally, dendritic cells in patients with AD secrete less IFN-α, and there is a reduction in natural killer (NK) cells (4, 19). Increased thymic stromal lymphopoietin (TSLP) acts as a critical alarmin in the skin primarily produced by keratinocytes in response to environmental stressors, including allergens and irritants. Once released, TSLP binds to its receptor on dendritic cells, particularly dermal dendritic cells, triggering their activation. This activation leads to the release of cytokines such as IL-4, IL-5, and IL-13, which are pivotal in driving the Th2-skewed immune response seen in AD (20).

#### 1.1.3 Dysbiosis of skin and intestinal flora

The skin microbiome—comprising bacteria, viruses, fungi, and arthropods—also undergoes alterations in patients with AD (21). Differences in microbial diversity and community composition have been observed between affected and unaffected skin of patients with AD. Genomic approaches have revealed characteristic, site-specific bacterial community structures, and shotgun metagenomics has shown that the overall microbial composition—including bacteria, fungi and virus—differs between patients with AD and controls across multiple skin sites (22). These microbial populations are dynamic and vary by body area and age (23). In healthy children, *Staphylococcus epidermidis*, *Staphylococcus lugdunensis*, *Staphylococcus hominis*, *Cutibacterium*, *Acinetobacter*, *Prevotella*, and *Corynebacterium* are more abundant than in children with AD (16, 24). Commensal coagulase-negative staphylococci inhibit the growth and biofilm formation of *Staphylococcus aureus* in healthy children, whereas *Staphylococcus aureus* predominates in patients experiencing AD flares (7). In over 90% of patients with AD, *Staphylococcus aureus* colonization is observed, compared to 15%–30% of healthy individuals (4, 25). Besides, greater colonization by *Staphylococcus aureus* is linked with increased inflammation and more severe disease (26). *Staphylococcus aureus* produces toxins that act as superantigens, promoting Th2 inflammation and enhancing the IgE-mediated response. The alpha-toxin of *Staphylococcus aureus* induces keratinocyte apoptosis, while the δ toxin increases mast cell degranulation (27).

A balanced and diverse skin microbiome can offer protection against pathogenic bacteria, while its disruption may contribute to AD development. For instance, Kennedy et al. (28) found that infants colonized with *Staphylococcus epidermidis* and *Staphylococcus cohnii* by two months of age had a significantly lower risk of developing eczema by one year, likely due to greater microbial diversity. Additionally, Nakatsuji et al. (29) reported that healthy individuals harbor significantly higher levels of coagulase-negative *Staphylococcus* species—such as specific strains of *Staphylococcus epidermidis* and *Staphylococcus hominis* with anti- *Staphylococcus aureus* properties—compared to patients with AD; and when these beneficial strains were applied to these patients, they effectively reduced *Staphylococcus aureus* colonization. Further, Byrd et al. (30) found that mild AD flares were associated with increased *Staphylococcus epidermidis* levels, whereas severe cases were dominated by *Staphylococcus aureus*.

The intestinal microbiota, composed of millions of microorganisms, plays a crucial role in immune system function and the regulation of inflammation. Recent research suggests that dysbiosis—an imbalance in this microbiota—may contribute to the development and exacerbation of AD. This connection is thought to be mediated through the interaction between intestinal bacteria and the immune system, which modulates the inflammatory response in the skin. Studies support this concept, showing that the gut microbiome not only regulates systemic immune responses but also directly impacts the skin immune function. Dysbiosis may disrupt the balance of T-helper cells, particularly the Th17/Treg cell axis, which is critical for controlling inflammation and maintaining skin homeostasis. Additionally, alterations in gut microbiota can lead to the production of pro-inflammatory cytokines and immune mediators, which may exacerbate skin inflammation and trigger or worsen AD. Certain bacterial strains in the gut, such as *Firmicutes* and *Bacteroidetes*, have been implicated in maintaining immune tolerance, while a decrease in microbial diversity—commonly associated with dysbiosis—is linked to increased susceptibility to inflammatory skin conditions like AD (31, 32).

The prevalence of AD is significantly higher in children (20%) compared to adults (3%) (1). Microbiome is considered one of the most influential factors contributing to this discrepancy. Recent metagenomic studies have elucidated significant age-related differences in the skin microbiome and gut microbiota, highlighting distinct microbial compositions between adults and children (33). In the skin microbiome, children exhibit a higher diversity of microbial communities compared to adults (33, 34). The increased diversity is attributed to the skin's developing immune system and environmental exposures, which influence microbial colonization patterns. Conversely, adults tend to have a more stable and less diverse skin microbiome, reflecting a mature immune system and established environmental interactions (35). The age-specific differences in the composition of skin commensals likely play a role in AD development since the commensals help defend against pathogens and maintain skin health at different development stages (36).

The higher abundance of *Staphylococcus aureus* in AD is independent of age group, ethnicity, and geographic location (37, 38); however, Shi et al. (36) demonstrated significant differences in the skin microbiome between pediatric and adult patients with AD by comparing the microbial patterns of 128 patients—59 young children (2–12 years), 13 adolescents (13–17 years), and 56 adults (18–62 years)—and healthy controls. Their analysis identified significant differences in microbial composition between young children and adolescent/adult patients with AD (beta diversity, ANOSIM *p* < 0.001)). In non-lesional AD skin, microbial diversity was significantly higher in young children than in adolescents/adults (alpha diversity, *p* = 0.036). However, in lesional skin, microbial diversity was significantly lower in both young children (*p* < 0.001) and adolescents/adults (*p* = 0.013). *Staphylococcus* was significantly more abundant in both lesional (*p* ≤ 0.012) and non-lesional skin of patients with AD compared with skin of healthy controls (*p* < 0.003).

Regarding the gut microbiota, children possess a less diverse microbial composition compared to adults (39). Moreover, and imbalance of the gut microbiome during early childhood precedes the onset of AD. By the age of three, the children gut microbiota resembles that of an adult, with three major microbial phyla—*Firmicutes*, *Bacteroidetes*, and *Actinobacteria*—becoming more prevalent (40, 41). This maturation process is influenced by multiple factors including diet, antibiotic usage, and environmental exposures (42, 43).

The environmental factors contribute to significant variability in metagenomic studies of children's gut microbiota (39, 44). However, as children age, their microbiota stabilizes, and the variations observed in younger children tend to diminish, ultimately resembling the more stable and diverse gut microbiota of adults. The gut microbiome of infants with AD shows a decreased relative abundance of *Bifidobacterium, Enterococcus, Clostridium, Lactobacillus paracasei,* and *Ruminococcaceae* (45–47). In contrast, gut colonization with *Staphylococcus*, *Clostridia*, and *Feacalibacterium prausnitzii* is more prevalent in AD infants (48, 49). Additionally, as in the skin, studies have noted higher counts of *Staphylococcus aureus* in fecal samples of AD children (33).

Wang et al. (50) found unique gut microbiome signatures in adult patients with moderate to severe AD in Southern Chinese populations. Their findings revealed a dominance of *Blautia, Butyricicoccus, Lachnoclostridium, Eubacterium halliigroup, Erysipelatoclostridium, Megasphaera, Oscillibacter*, and *Flavonifractor*. However, a recent systematic review on gut dysbiosis and adult AD did not find global differences in gut microbiota between adults with AD and healthy adults. Nevertheless, specific bacterial taxa, including *Bacteroides*, *Escherichia-Shigella* and *Clostridium* were more characteristic of the fecal microbiota in adults with AD (51). Furthermore, a higher prevalence of *Clostridia* and *Enterobacteriaceae* species has been detected in both children and adults with AD (44).

These findings underscore the dynamic nature of the gut microbiota and its potential implications in AD pathogenesis across different age groups.

#### 1.1.4 Associated infections

Children with AD are particularly susceptible to infections, which can trigger or worsen AD flares. As such, it is important to recognize infection-related flare-ups and understand the appropriate management strategies. Infections typically begin in early childhood, with *Staphylococcus aureus* overgrowth on untreated AD lesions being common. As children grow, the spectrum of infections broadens, including widespread molluscum contagiosum infections in toddlers and school-aged children, folliculitis and impetigo in scholars, and a higher prevalence of warts in pre-teens and teens (52).

Huang et al. (44) compared 86,969 pediatric patients with AD to 116,564 matched controls and found that children with AD had significantly higher odds of developing various skin infections. These included methicillin-resistant *Staphylococcus aureus* (MRSA) (OR, 3.76), varicella (OR, 2.12), and herpesvirus infections (OR, 2.91) compared to the matched controls.

Additionally, Ren and Silverberg documented that children with AD had higher rates of skin infections in emergency department visits compared to those without AD (5.15% vs. 2.48%). They also found that AD was associated with significantly higher odds of skin infection (OR 2.23, 95% CI 2.16–2.31). The infections with higher adjusted odds ratios included eczema herpeticum (OR 12,95, 95% CI 10.72–15.66), impetigo (OR 6.64, 95% CI 6.29–7.00), molluscum contagiosum (OR 4.58, 95% CI 4.18–5.02), and erysipelas (OR 3.63, 95% CI 2.63–5.01). Other infections with increased odds in children with AD included carbuncle/furuncle, cellulitis, MRSA and non-MRSA infections, cutaneous warts, herpes simplex and zoster viruses, dermatophytosis and candidiasis (53).

A notable aspect is the comparison of infectious agents between pediatric and adult patients. In AD cutaneous infections show age-related differences. Molluscum contagiosum and impetigo are more common in children with AD. While *Staphylococcus aureus*, Herpes Simplex Virus, Human papillomavirus and Coxsackie virus are slightly more prevalent in children, the difference is less significant. In contrast, *Malassezia spp.* and *Candida spp*. infections are more frequent in adults, especially those with chronic or seborrheic-like AD. Table 1 summarizes infectious agents associated with an increased frequency in patients with AD, along with a comparison between adults and children (54–57).

**Table 1:** Primary cutaneous infectious agents in patients with atopic dermatitis.

Pathogen	Disease	Clinical features	Diagnostic test	Treatment	Hospitalization criteria	Pediatric vs. adult
*Staphylococcus aureus*	Non-bullous impetigo Bullous impetigo Other less frequent: Folliculitis, furunculosis, cellulitis, erysipelas, dactylitis	Erythema, warmth, tenderness, edema, and a serous discharge that, upon drying, forms a meliceric crust Large fluid-filled bullae that rupture easily, leaving a moist red base	Skin cultures (especially if MRSA is suspected)	Topical antibiotics; if systemic signs are present, use systemic antibiotics*	Persistent fever, severe skin infection, suspected organ failure.	Pediatric patients have higher rates of colonization and infection. Adults with AD are more likely to develop impetigo in the setting of severe disease or immunosuppression.
Streptococcus pyogenes	Non-bullous impetigo Other less frequent: cellulitis, erysipela, ecthyma and dactylitis.	Erythema, warmth, tenderness, edema, and a serous discharge that, upon drying, forms a meliceric crust	Skin and swab cultures	Topical antibiotics, for severe or disseminated infections, systemic treatment is recommended*	Persistent fever, severe skin infection, suspected organ failure.	Highly prevalent in children.
Herpes simplex virus	Eczema herpeticum	Pruritic, painful vesicles, ulcerations, and widespread crusts exacerbated by scratching	PCR or Tzanck test.	Acyclovir, valacyclovir or famciclovir* Oral acyclovir should be restricted to the treatment of mild disease	Moderate to severe EH. Intravenous acyclovir is indicated.	Viral infections, such as HSV, are more prevalent in pediatric patients and can lead to severe complications
Coxsackie virus	Eczema Coxsackie	Hand, foot, and mouth disease, characterized by oral ulcers and papules on the hands and feet, followed by systemic symptoms such as fever, malaise, and sore throat	PCR for Coxsackie A16 and other enteroviruses	Supportive treatment for symptoms	Rarely; patients develop complications such as aseptic meningitis, encephalitis, seizures, myopericarditis, or heart failure which warrant hospitalization.	Coxsackie virus is more commonly associated with pediatric AD due to higher exposure rates, while adults are less likely to develop infections unless immunocompromised
Molluscum contagiosum virus	Eczema molluscatum	Small, pearl-like papules that may have a central umbilication	Not necessary; if in doubt histopathology of lesions	Curettage or chemical irritants	Not require In disseminated cases, an underlying immunodeficiency must be ruled out	Molluscum contagiosum occurs mainly in pediatric AD.
Vaccinia virus	Eczema vaccinatum	Vesicles, crusts, and associated fever	PCR of vaccinia virus	Vaccinia immune globulin Cidofovir Brincidofovir	All cases should be hospitalized. Patients should be isolated until a case of smallpox is ruled out. Infection-control precautions should be used to prevent secondary transmission and nosocomial infection.	Most cases occur in adults
Human papillomavirus	Warts	Small, raised, keratotic papules with tiny black dots in the center	Not necessary; if in doubt PCR of HPV	Cryotherapy, chemical irritants, immunotherapy	Not required In disseminated or recalcitrant cases, an underlying immunodeficiency must be ruled out	HPV infection is slightly more frequent in pediatric patients with AD.
*Malassezia spp.*	Head and neck dermatitis	Eczematous plaques in seborrheic areas	Prick test, specific immunoglobulin E test, or atopy patch test	Topical antifungals: ketoconazole or ciclopirox olamine Systemic antifungals: itraconazole or fluconazole	Not required In severe or recalcitrant cases, an underlying immunodeficiency must be ruled out	Malassezia and other fungal infections are less common in pediatric AD but occur more frequently in adults with AD.

MRSA, methicillin-resistant *Staphylococcus aureus*; PCR, polymerase chain reaction; HPV, human papillomavirus.

Recent research also indicates that AD is associated with higher rates of extracutaneous infections, such as respiratory and urinary tract infections, in both adult and pediatric populations (58). In this regard, Huang et al. (44) found increased odds of several infections in pediatric patients with AD, including influenza (OR,1.40), pneumonia (OR, 1.52), bronchitis (OR, 1.42), gastroenteritis (OR, 1.70), urinary tract infections (OR, 1.38), otitis media (OR, 1.43), streptococcal pharyngitis (OR, 1.29), and sinusitis (OR, 1.52), compared to matched controls. However, further investigation is needed to elucidate the underlying mechanisms behind these extracutaneous and systemic infections in AD.

## 2 Bacterial infections

Bacteria constitute approximately 70% of the normal skin microbiome. In children with AD, certain infections occur more frequently, including impetigo, erysipelas, cellulitis, cutaneous abscesses, and folliculitis (59, 60).

### 2.1 Staphylococcus aureus

*Staphylococcus aureus* is the most common bacterial pathogen in children with AD, accounting for around 40% of infections in this population (60–63). It is a Gram-positive opportunistic bacterium capable of causing both superficial and invasive infections. While *Staphylococcus aureus* colonizes the skin in only 15%–30% of the general population (64), its prevalence is significantly higher in patients with AD, especially those with moderate to severe disease. In such patients, the likelihood of colonization correlates with disease severity (64). In contrast to healthy children, *Staphylococcus aureus* is found in 70%–90% of the skin with active dermatitis, 39% of unaffected skin, and 62% of nasal passages in children with AD (65).

This increased colonization is attributed to alterations in filaggrin, which lead to a decreased NMF and lower skin acidification, enabling greater expression of bacterial virulence genes including enterotoxins, phenol-soluble modulins, factor B, and alpha-hemolysin, which enhance bacterial adhesion to keratinocytes (60).

**Figure 2:**
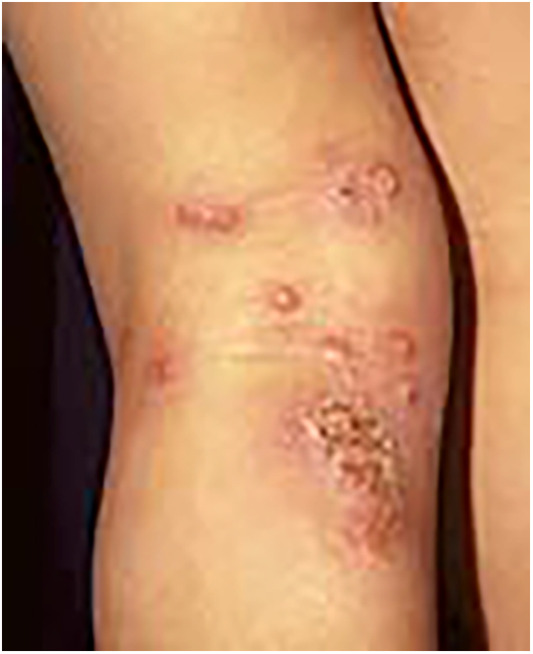
Impetiginized atopic dermatitis lesions.

Non-bullous impetigo is the most common clinical manifestation of *Staphylococcus aureus* infection in pediatric patients with AD, presenting with erythema, warmth, tenderness, localized skin edema, and a serous discharge that, upon drying, leaves a meliceric crust (Figure 2) (61). *Staphylococcus aureus* infection seldom appears as bullous impetigo, which typically appears as clusters of vesicles that rapidly progress to flaccid superficial bullae. These bullae easily rupture, leaving moist, red erosions surrounded by a scaly collarette of blister roof. In this context, bullous impetigo is often misdiagnosed as an acute AD flare, scabies, varicella, or other conditions (62). Laboratory tests in affected patients may reveal elevated acute phase reactants like C-reactive protein (CRP) or erythrocyte sedimentation rate (ESR). If left untreated, these infections can lead to bacteremia, dissemination to other organs, and staphylococcal scalded skin syndrome (63).

### 2.2 Streptococcus pyogenes

AD is also associated with a higher incidence of *Streptococcus pyogenes* infections, with 24% of AD children affected compared to 17% of the healthy children. *Streptococcus pyogenes* can cause both cutaneous (impetigo) and extracutaneous infections, such as pharyngotonsillitis (66). Cutaneous lesions may appear as erosions with scalloped edges that resemble eczema herpeticum. *Streptococcus pyogenes* infections may occur alone or in combination with *Staphylococcus aureus*, and the two can be clinically indistinguishable (67).

## 3 Viral infections

Viral infections are less common than bacterial infections in children with AD. The most frequently encountered viral infections include herpetic eczema, coxsackie eczema, and molluscum contagiosum. In addition, we mention evidence of association od AD with human papillomavirus and SARS-CoV2.

### 3.1 Eczema herpeticum

**Figure 3:**
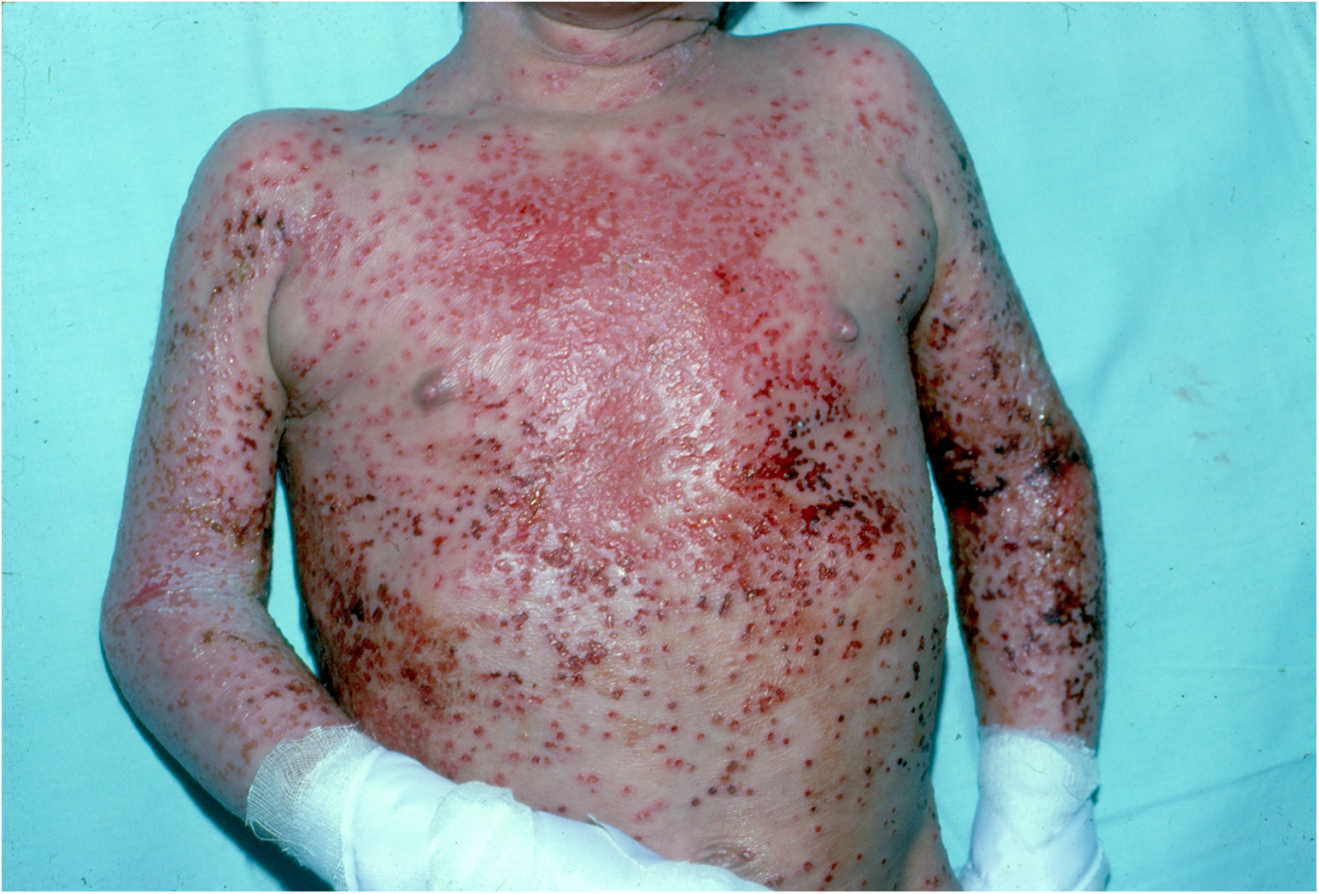
Patient with multiple vesicles and crusts in eczema herpeticum.

**Figure 4:**
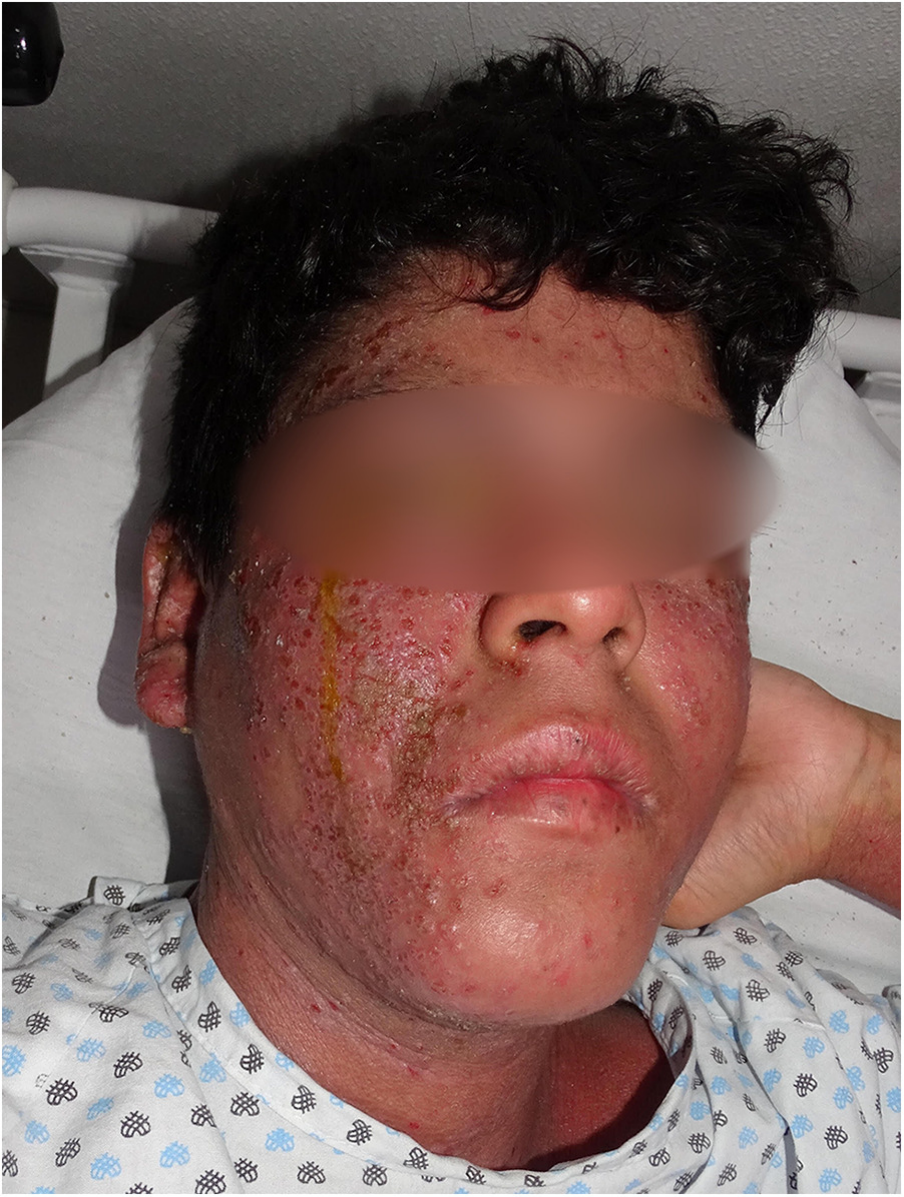
A 14-year-old male adolescent patient with impetiginized eczema herpeticum.

Eczema herpeticum, caused by the herpes simplex virus, can spread rapidly and poses a serious, life-threatening risk. Although only 3% of children with AD develop herpetic eczema, it accounts for up to 34% AD-related hospitalizations (68, 69). Initial studies indicated that the R501X mutation in the gene encoding filaggrin (OMIM *135940, FLG), one of the strongest genetic predictors of AD, significantly increases the risk of developing eczema herpeticum in both European and African ancestry populations. This suggests that a defective skin barrier plays a role in the development of this severe condition (70). More recently, deficiencies in claudins and overexpression of indoleamine 2,3-dioxygenase (IDO1) have been observed (68). Clinically, it presents with pruritic, painful vesicles, ulcerations, and widespread crusts exacerbated by scratching Figure 3. The condition typically occurs in children with more severe forms of AD and is often associated with impetiginous coinfection (Figure 4) (68, 71, 72).

The increased risk of *Staphylococcus aureus* infection may be due to heavy colonization of *Staphylococcus aureus* in these children and to the production of α-toxins that can promote viral replication in skin cells (71).

Systemic symptoms such as fever, malaise, and lymphadenopathy are common, and complications may involve other organs, leading to keratoconjunctivitis, meningoencephalitis, and, in severe cases, septic shock (70).

### 3.2 Coxsackie eczema

**Figure 5:**
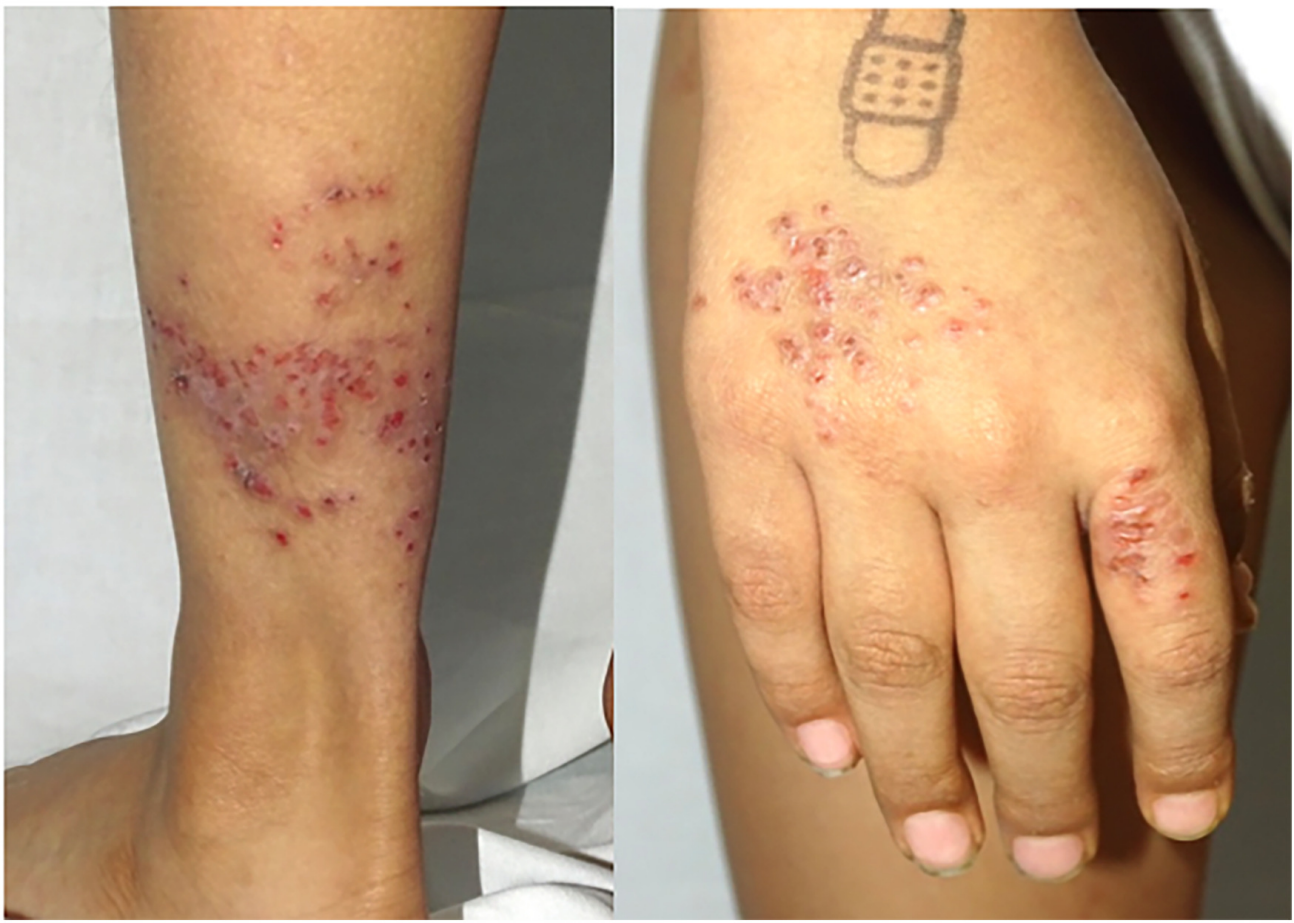
Coxsackie eczema almost indistinguishable from eczema herpeticum.

Although specific percentages regarding the incidence of coxsackie eczema in children with AD remain unreported, existing research suggests a significant association between the two conditions. One study found that 55% of coxsackie eczema cases occurred in children with underlying AD (73). Coxsackie eczema is characterized by disseminated vesicles and ulcerations. It is an infection caused by enteroviruses, with coxsackie A6 being the most common strain. It may initially manifest as hand, foot, and mouth disease, featuring oral ulcers and papules on the hands and feet, followed by systemic symptoms like fever, malaise, and sore throat. Although there is no specific evidence that coxsackie eczema is more frequent in AD children, lesions tend to be more widespread in this population, and it can be easily confused with herpetic eczema (Figure 5) (65).

### 3.3 Molluscum contagiosum

Children with AD have a 13% higher risk of contracting molluscum contagiosum infections compared to healthy children (74). Besides, studies have shown that children with a history of AD are more likely to have a higher number of molluscum contagiosum lesions, as well as a higher prevalence of molluscum dermatitis (75). Additionally, in children with pre-existing AD, molluscum contagiosum can exacerbate the disease, leading to more widespread lesions. In children, the risk of exacerbation appears to be highest when molluscum contagiosum lesions develop on intertriginous or flexural areas (76).

**Figure 6:**
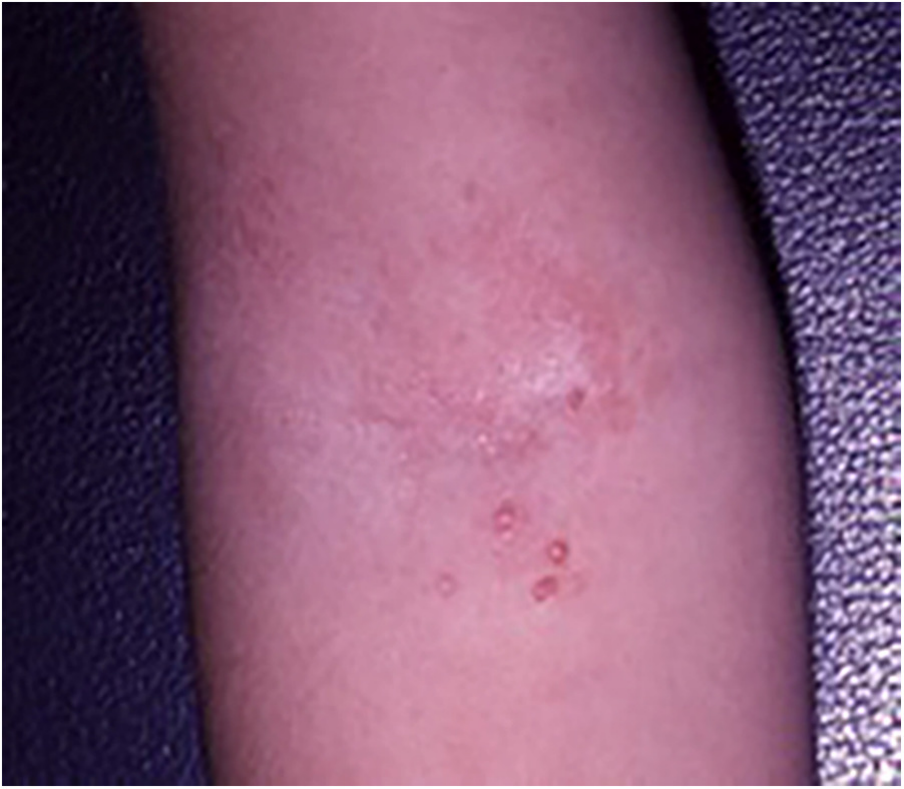
Typical pearl-like papules in molluscum eczema.

Molluscum contagiosum is a virus, member of the poxvirus family, and tends to be more widespread in those with AD, particularly in areas prone to scratching. It is characterized by small, pearl-like papules that may have a central umbilication (Figure 6). The infection can cause associated eczema in the area. Constant scratching can lead to autoinoculation and further spread of the lesions. While eczema associated with molluscum contagiosum is not typically severe, it can be cosmetically significant (76).

### 3.4 Human papillomavirus infection

There are conflicting reports about the risk for acquisition of warts in children with AD. While some studies have reported a lower incidence of warts in patients with AD, more recent research with a larger sample size found a higher prevalence of warts in children with AD and other atopic disorders (7%), though a slightly lower prevalence in patients with AD alone (2%) (56).

### 3.5 SARS-CoV-2 infection

The relationship between SARS-CoV-2 infection and AD is bidirectional: patients with AD have an increased risk of SARS-CoV-2 infection, while COVID-19 can trigger new-onset or exacerbation of AD. However, most studies on this topic have focused on adults, with limited data available for pediatric populations (77, 78).

A large epidemiological study of 435,019 adult patients conducted by Patrick et al. (79) found that AD was significantly associated with an increased risk of SARS-CoV-2 infection (OR 1.48, 95%CI 1.06–20.6; *p* = 0.020), but a decreased risk of requiring mechanical ventilation (OR 0.22, 95%CI 0.11–0.47; *p* = 0.00008). Additionally, increased disease activity following COVID-19 infection or SARS-CoV-2 vaccination was observed in a minority of patients with AD (12/176; 6.8%) (80).

The association between AD and increased susceptibility to SARS-CoV-2 infection in children remains unclear. A retrospective chart analysis was conducted in Southern Brooklyn, New York, an area of high COVID-19 incidence. The study included 677 patients diagnosed with AD, non-eczema dermatitis, asthma, or allergy, alongside1505 healthy controls. Participants were tested using COVID-19 rapid antigen, SARS-CoV-2 IgG antibody, or SARS CoV-2 IgM antibody. The results showed that within the tested community, children with AD or allergic disorders had similar rates of COVID-19 infection compared to heatlhy children (81).

Elevated levels of IL-4 and IL-13, key cytokines associated with AD, have been linked to more severe COVID-19 outcomes. Dupilumab, an IL-4 and IL-13 inhibitor used in AD treatment, has shown promise in reducing the severity of both AD and COVID-19 by modulating these cytokines (79).

A large cohort study including 617,964 COVID-19 patients and 1,796,174 matched-control cases demonstrated a significant increase in new-onset AD among patients with a history of SARS-CoV-2 infection compared to those with negative serology. The highest risk difference was observed in the pediatric population under 18 years of age, with those having prior COVID-19 infection exhibiting a 33% increased risk of developing AD compared to controls (80).

One study investigated the link between COVID-19 and AD by analyzing large-scale genetic, transcriptomic, and epigenetic data. The findings suggest that epigenetic modifications and transcriptional regulation contribute to COVID-19-associated onset and worsening of AD. Notably, LMAN2 was identified as a key molecule linking viral infection to immune-mediated inflammatory diseases. However, further research—particularly in pediatric populations—is required to fully elucidate these connections and optimize treatment strategies (81).

## 4 Fungal infections

The diversity of fungi is greater in active lesions of AD than in non-lesional skin (82, 83). Yeasts from the *Malassezia* family are part of the normal microbiota, primarily found near the openings of sebaceous glands and in the upper parts of hair follicles. Colonization occurs in 100% of patients with AD, compared to 10%–78% in healthy children (58, 84). In pediatric patients with AD, an overgrowth of *Malassezia spp*. has been observed; *Malassezia globosa* and *Malassezia restricta* are present in both healthy individuals and children with AD. However, a higher quantity of *Malassezia dermatitis* and *Malassezia sympodialis* has been found in children with AD, with no distinction between affected and unaffected skin (58). Additionally, a large population study showed more than 40% of children with seborrheic dermatitis during early childhood will develop AD later on, suggesting early sensitization of seborrheic skin may result in the onset of AD (85). Elevated levels of total IgE and specific IgE against *Malassezia* have been observed in children with AD, leading to speculation that *Malassezia* in the sweat of these children may act as an allergen, contributing to inflammation (86–88).

Head and neck dermatitis is a subtype of AD that affects the seborrheic areas and is more common in children. Current evidence implicates fungi, particularly *Malassezia spp.* in its pathogenesis. Clinically, it presents as eczematous plaques that consistently affect the forehead, eyelids, perioral region, and neck. This condition has been primarily associated with patients with AD treated with dupilumab, although its underlying mechanisms remain incompletely understood (84).

Although *Candida spp*. has not been directly associated with a dermatosis, an increased prevalence of *Candida spp*. has been identified in children with AD compared to healthy children. Candida commonly colonizes the oral, gastrointestinal, and urogenital mucosa, affecting 50%–75% of individuals. *Candida albicans* has been reported to induce alterations in keratinocytes that facilitate interactions with antigen-presenting cells in patients with AD (88, 89).

Children with AD who develop chronic dermatophyte infections often experience more severe and persistent symptoms (89). Chronic dermatophyte infections are more prevalent in children with AD, and their management tends to be more challenging compared to pediatric patients without AD (89). While antifungal treatments may provide some relief, a more comprehensive understanding of the relationship between dermatophyte infections and the progression of AD is still needed (88).

## 5 Diagnosis

Clinical data is typically sufficient to guide diagnosis. Microbiological skin cultures are generally not recommended unless there is suspicion of MRSA to determine antibiotic sensitivity and guide treatment. If a systemic *Streptococcus pyogenes* infection is suspected, a throat swab culture can be performed due to its association with pharyngotonsillitis, or a serology test for anti-streptolysins may be conducted. For cutaneous *Streptococcus pyogenes* infection, a skin culture could be performed (3, 61).

In cases where herpetic or coxsackie eczema are suspected but the presentation is unclear, PCR testing of lesion exudate is recommended for confirmation of HHV-1 or Coxsackie virus. If PCR is unavailable, a scraping of the lesion for a Tzanck test to identify multinucleated giant cells can be performed (56).

For fungal associated infections, since *Malassezia spp.* and *Candida spp.* are commensal organisms, routine smears and molecular testing are not recommended. In these cases, sensitization tests such as prick tests, specific immunoglobulin E tests, or atopy patch tests are more useful (89).

## 6 Treatments for infectious agents

Bacterial and viral infections in AD often emerge suddenly and are more prevalent in severe cases. In contrast, fungal infections typically develop more gradually and may not be immediately obvious. These infections frequently worsen the symptoms of AD, highlighting the importance of prompt and effective treatment to manage both the infections and the underlying condition (90, 91).

Active lesions of AD require topical corticosteroids as the first-line treatment. In patients with active *Staphylococcus aureus* infections (rather than colonization), the addition of topical antibiotics may be considered, although their use should be limited to short periods to prevent bacterial resistance (92). Some guidelines advise against the use of topical antibiotics due to their limited efficacy compared to corticosteroids alone (90, 93). In cases of disseminated infections or those with systemic involvement, systemic antibiotics are recommended (94).

Studies indicate that using antibiotics to decolonize patients with *Staphylococcus aureus* is ineffective in preventing exacerbations (16). Consequently, the widespread use of systemic antibiotics or routine decolonization is not recommended, as these approaches can disrupt the skin microbiome and contribute to increased antibiotic resistance (95). Additionally, prophylactic antibiotics have not demonstrated any benefit in reducing inflammation in patients with AD in the absence of active infection (94).

*Staphylococcus aureus* infections often require antibiotic treatment, with choices guided by methicillin sensitivity and resistance patterns. For mild infections suspected to involve methicillin-sensitive *Staphylococcus aureus*, recommended options include amoxicillin, cephalexin, doxycycline, minocycline, or clindamycin. In children, MRSA is more prevalent, and multiple resistance genes are often present, necessitating careful antibiotic selection (93). If MRSA is suspected, linezolid or trimethoprim-sulfamethoxazole should be considered. For severe infections, both methicillin-sensitive and methicillin-resistant strains should be covered using a combination of vancomycin, linezolid, teicoplanin, or daptomycin, alongside an anti-staphylococcal beta-lactam antibiotic, with intravenous administration recommended. The duration of antibiotic therapy typically ranges from 7 to 14 days, adjusted based on local resistance patterns (90). A recent systematic review advises against the empirical use of beta-lactams, erythromycin, clindamycin, or fusidic acid in patients with AD due to high microbial resistance (94).

Decolonization can be beneficial for patients with recurrent exacerbations, particularly through nasal decolonization with topical mupirocin for 5 days. Treatment should also extend to family members and pets to reduce household reservoirs of infection. However, complete eradication remains difficult, as recurrent infections are often associated with persistent colonization within households (95).

For localized skin infections caused by *Streptococcus pyogenes,* topical antibiotics like fusidic acid or mupirocin can be used. For more severe or disseminated infections, systemic treatment with penicillin is recommended. Allergic patients may be treated with macrolides. Severe infections can be managed intravenously with vancomycin or clindamycin (96, 97).

In cases of suspected eczema herpeticum, empirical treatment should be initiated immediately. If there is dissemination to more than one segment, hospitalization and initial intravenous therapy are warranted, with acyclovir, valacyclovir, or famciclovir as the preferred medications. Topical antivirals are not effective (98).

Coxsackie eczema is generally benign and treated supportively, with topical corticosteroids for intense itching. Treatment for molluscum contagiosum can vary. The most effective method is curettage but may be poorly tolerated by children. Other alternatives include cryotherapy or chemical irritants. A common effective option for children is the nightly topical application of 10% KOH (99).

Head and neck dermatitis is primarily managed with topical antifungals, such as ketoconazole or ciclopirox olamine. In more severe or refractory cases, systemic antifungal therapy, including itraconazole or fluconazole, may be considered as an alternative (84).

### 6.1 Prevention of cutaneous infections by restoring the skin barrier and treating AD

#### 6.1.1 Emollients and moisturizers

The daily and frequent application of emollients and moisturizers is crucial for repairing and maintaining the skin barrier, thereby reducing the risk of infections. Their use has been shown to decrease the quantity of *Staphylococcus aureus* on the skin (100). A daily bath or shower with lukewarm water, followed by gentle drying, is recommended. Moisturizers should be applied multiple times a day to keep the skin hydrated. Ointments are generally more effective than creams and lotions for maintaining skin hydration, although they may not always be well tolerated by all patients (101). Petrolatum is recommended, as it helps maintain barrier function and supports normal skin microbiota. However, in excessively hot or humid environments, its use may be discouraged due to its occlusive nature (101).

#### 6.1.2 Treating *staphyloccocus aureus* colonization

Diluted sodium hypochlorite baths (0.005%) have been shown to aid in disease control, particularly in patients already undergoing anti-inflammatory treatments, further reducing the burden of *Staphylococcus aureus* (102). These baths are typically recommended once or twice a week, using commercially available bleach (5%–6% concentration). The bleach is diluted at a ratio of 1–2 ml per L of water. A meta-analysis found that this treatment improves the severity of AD in moderate to severe cases without significant adverse effects. However, despite its clinical benefits, the same meta-analysis found no significant reduction in *Staphylococcus aureus* burden. Thus, while chlorine baths have a beneficial anti-inflammatory effect, their overall impact on the skin microbiome remains unclear (103).

Bacteriotherapy is an emerging approach for treating AD by restoring microbial balance and reducing *Staphylococcus aureus* colonization, a key factor in inflammation and barrier dysfunction. This strategy involves the use of probiotics, bacterial lysates, enzymes, and microbiome transplants to promote a healthier skin microbiome (104).

Current evidence suggests that oral prebiotics and probiotics do not significantly impact AD severity, as measured by SCORAD. However, studies indicate that the topical application of certain *Lactobacillus* species (e.g., *Lactobacillus plantarum* and *Lactobacillus salivarius*) can reduce *Staphylococcus aureus* colonization. though this has not yet translated into improved AD lesions or reduced corticosteroid use (30).

Recent studies highlight the potential benefits of antimicrobial peptides (AMPs) produced by coagulase-negative commensal staphylococci, such as *Staphylococcus epidermidis, Staphylococcus lugdunensis* and *Staphylococcus hominis*, within the human microbiome (105). These bacteria produce unique peptides and lantibiotics, that enhance skin defense by selectively targeting and eliminating pathogenic bacteria, such as *Staphylococcus aureus*, while preserving beneficial microbes. By synergizing with the host's endogenous AMPs, these bacterial peptides strengthen the skin antimicrobial barrier, maintain microbial balance, and help prevent infections (106).

Furthermore, studies in both animal models and patients with AD have shown promising therapeutic benefits, particularly by targeting *Staphylococcus aureus*, leading to clinical improvements in AD. While preliminary findings are encouraging, larger clinical trials are needed to confirm efficacy and long-term effects (107).

Newer therapies, such as endolysins, are being explored for AD management due to their ability to selectively target *Staphylococcus aureus* by cleaving peptidoglycan bonds in the bacterial cell wall. These lysins, derived from bacteriophages, offer a novel antimicrobial approach with high specificity (108).

Niclosamide, a traditional anthelmintic agent, has recently being investigated for its potential role in treating AD, particularly through its effects on microbial dysbiosis. Topical niclosamide (ATx201) has shown promise in reducing *Staphylococcus aureus* colonization. In a Phase 2 randomized, double-blind, placebo-controlled trial, ATx201 significantly decreased *Staphylococcus aureus* burden while enhancing skin microbiome diversity in patients with AD. This shift towards a more balanced microbiota is associated with improved skin health and reduced inflammation, highlighting niclosamidés potential as an adjunct therapy for AD (106).

#### 6.1.3 Anti-inflammatory therapies

Topical anti-inflammatory treatments, such as corticosteroids, crisaborole, and calcineurin inhibitors, are effective for reducing inflammation, restoring barrier function, and decreasing *Staphylococcus aureus* colonization. Controlling inflammation is essential for preventing infections, as it is a major risk factor for skin infections in patients with AD (109).

Dupilumab, a monoclonal antibody that targets and neutralizes IL-4 and IL-13, has been shown to significantly reduce pruritus, inflammation, and *Staphylococcus aureus* colonization. A clinical study demonstrated that after 32 weeks of treatment, dupilumab induced significant changes in the microbiome of skin lesions, by reducing *Staphylococcus aureus* colonization in 75% (110). Dupilumab is approved for use in pediatric patients aged 6 months and older. Other monoclonal antibodies with similar efficacy, such as tralokinumab and lebrikizumab, are approved for use in patients aged 12 years and older (104).

Other treatments, such as Janus kinase (JAK) inhibitors, both topical and oral, are currently in various stages of clinical trials for pediatric patients. Baricitinib, administered orally, and topical ruxolitinib have been approved for the treatment of atopic dermatitis (AD). Baricitinib is approved for daily oral use in moderate to severe cases in children aged 2 years and older, although its efficacy has primarily been demonstrated in children over 10 years of age. Topical ruxolitinib (1.5%) is approved for short-term treatment of mild to moderate AD in patients aged 12 years and older (111, 112). Abrocitinib is approved for children aged 12 years and older, with clinical outcomes similar to those of other JAK inhibitors. Upadacitinib shows promise, demonstrating superior efficacy compared to dupilumab after 4 months of treatment; however, additional studies are needed to further establish its safety and efficacy in children (113). Topical delgocitinib has been approved in Japan for the treatment of moderate to severe AD in children aged 2 years and older. These therapies provide benefits for patients who do not achieve adequate control with other treatments and have not been associated with an increased risk of infections (114).

Narrow-band UVB (NB-UVB) phototherapy is an effective and well-tolerated treatment for moderate to severe AD, particularly in patients unresponsive to topical therapies. It modulates immune responses by inducing apoptosis of activated T cells, reducing pro-inflammatory cytokines such as TNF-α and IL-4, and promoting the production of anti-inflammatory cytokines. Clinical studies report a 60%–80% improvement in AD symptoms, including erythema, pruritus, and scaling, with sustained benefits and fewer side effects than broader UV spectra (115). NB-UVB also reduces *Staphylococcus aureus* colonization and its production of superantigens (107). While psoralen plus UVA (PUVA) is an alternative for severe AD, it carries a higher risk of long-term skin damage and carcinogenesis. Phototherapy is frequently combined with topical or systemic treatments to enhance efficacy, providing a viable option with a lower risk profile than prolonged immunosuppressive therapy (115).

#### 6.1.4 Treating pruritus

Pruritus and its consequent scratching significantly contribute to skin damage, making its control a primary treatment goal. Conventional antihistamines have little direct effect on pruritus, as the pathways involved in AD are not primarily mediated by histamine. Their utility lies mainly in their sedative effects, which is why they are often used at night. In contrast, medications that block the IL-4/IL-13 pathway, such as dupilumab, markedly improve these symptoms. Additionally, blocking the IL-31 pathway with drugs like nemolizumab or JAK inhibitors has shown significant antipruritic effects (116).

## 7 Conclusions

Patients with AD are more susceptible to frequent and severe infections than the general population A key factor contributing to this increased susceptibility is the presence of skin barrier defects, which lead to dysfunctional immune responses and pathogen invasion, resulting in inflammation and exacerbation of AD lesions. An altered skin microbiome further contributes by facilitating the overcolonization of potential pathogens, such as *Staphylococcus aureus* and *Malassezia spp*. These microorganisms are major contributors to both cutaneous an exracutaneous infections, further aggravating the condition. During AD flare-ups, infections should be considered potential triggers, as identifying and addressing the primary infectious agents can help prevent complications and reduce disease severity. Finally, infection prevention in AD should focus on two main strategies: restoring the skin barrier to prevent pathogen invasion and modulating the Th2 inflammatory response through targeted pharmacological interventions. This dual approach may help mitigate infections and alleviate associated complications (117).

## References

[1] Nutten S. Atopic dermatitis: global epidemiology and risk factors. Ann Nutr Metab. (2015) 66(Suppl 1):8–16. 10.1159/000370220 25925336

[2] Silverberg JI Barbarot S Gadkari A Simpson EL Weidinger S Mina-Osorio P Atopic dermatitis in the pediatric population: a cross-sectional, international epidemiologic study. Ann Allergy Asthma Immunol. (2021) 126(4):417–428.e2. 10.1016/j.anai.2020.12.020 33421555

[3] Garg N Silverberg JI. Epidemiology of childhood atopic dermatitis. Clin Dermatol. (2015) 33(3):281–8. 10.1016/j.clindermatol.2014.12.004 25889128

[4] Ong PY Leung DY. Bacterial and viral infections in atopic dermatitis: a comprehensive review. Clin Rev Allergy Immunol. (2016) 51(3):329–37. 10.1007/s12016-016-8548-5 27377298

[5] Narla S Silverberg JI. Association between atopic dermatitis and serious cutaneous, multiorgan, and systemic infections in US adults. Ann Allergy Asthma Immunol. (2018) 120(1):66–72.e11. 10.1016/j.anai.2017.10.019 29273131 PMC5745030

[6] Ahmad-Nejad MDS Breuer K Klotz M Werfel T Herz U Heeg K. The toll-like receptor 2 R753Q polymorphism defines a subgroup of patients with atopic dermatitis having severe phenotype. J Allergy Clin Immunol. (2004) 113(3):565–7. 10.1016/j.jaci.2003.12.583 15007364

[7] Rippke F Schreiner V Doering T Maibach HI. Stratum corneum pH in atopic dermatitis: impact on skin barrier function and colonization with *Staphylococcus aureus*. Am J Clin Dermatol. (2004) 5(4):217–23. 10.2165/00128071-200405040-00002 15301569

[8] Cabanillas B Novak N. Atopic dermatitis and filaggrin. Curr Opin Immunol. (2016) 42:1–8. 10.1016/j.coi.2016.05.002 27206013

[9] Furue M. Regulation of filaggrin, loricrin, and involucrin by IL-4, IL-13, IL-17A, IL-22, AHR, and NRF2: pathogenic implications in atopic dermatitis. Int J Mol Sci. (2020) 21(15):5382. 10.3390/ijms21155382 32751111 PMC7432778

[10] Lopes C Rocha L Sokhatska O Soares J Tavaria F Correia O Filaggrin polymorphism Pro478Ser is associated with the severity of atopic dermatitis and colonization by staphylococcal aureus. J Investig Allergol Clin Immunol. (2016) 26(1):70–2. 10.18176/jiaci.0017 27012026

[11] Wolk K Mitsui H Witte K Gellrich S Gulati N Humme D Deficient cutaneous antibacterial competence in cutaneous T-cell lymphomas: role of Th2-mediated biased Th17 function. Clin Cancer Res. (2014) 20(21):5507–16. 10.1158/1078-0432.CCR-14-0707 25212608

[12] Hata TR Gallo RL. Antimicrobial peptides, skin infections, and atopic dermatitis. Semin Cutan Med Surg. (2008) 27(2):144–50. 10.1016/j.sder.2008.04.002 18620136 PMC2546601

[13] Langan SM Abuabara K Henrickson SE Hoffstad O Margolis DJ. Increased risk of cutaneous and systemic infections in atopic dermatitis—a cohort study. J Invest Dermatol. (2017) 137(6):1375–7. 10.1016/j.jid.2017.01.030 28202403 PMC5660507

[14] Ong PY Ohtake T Brandt C Strickland I Boguniewicz M Ganz T Endogenous antimicrobial peptides and skin infections in atopic dermatitis. N Engl J Med. (2002) 347(15):1151–60. 10.1056/NEJMoa021481 12374875

[15] Kasperkiewicz M Schmidt E Ludwig RJ Zillikens D. Targeting IgE antibodies by immunoadsorption in atopic dermatitis. Front Immunol. (2018) 9:254. 10.3389/fimmu.2018.00254 29520268 PMC5827554

[16] Wollenberg A Thomsen SF Lacour JP Jaumont X Lazarewicz S. Targeting immunoglobulin E in atopic dermatitis: a review of the existing evidence. World Allergy Organ J. (2021) 14(3):100519. 10.1016/j.waojou.2021.100519 33815652 PMC8005850

[17] Holm JG Agner T Clausen ML Thomsen SF. Determinants of disease severity among patients with atopic dermatitis: association with components of the atopic march. Arch Dermatol Res. (2019) 311(3):173–82. 10.1007/s00403-019-01895-z 30770978

[18] Jinnestal CL Belfrage E Back O Sonesson A. Skin barrier impairment correlates with cutaneous *Staphylococcus aureus* colonization and sensitization to skin-associated microbial antigens in adult patients with atopic dermatitis. Int J Dermatol. (2014) 53(1):27–33. 10.1111/ijd.12198 23879225

[19] Mack MR Brestoff JR Berrien-Elliott MM Trier AM Yang TB McCullen M. Blood natural killer cell deficiency reveals an immunotherapy strategy for atopic dermatitis. Sci Transl Med. (2020) 12(532):eaay1005. 10.1126/scitranslmed.aay1005 32102931 PMC7433875

[20] Leyva-Castillo JM Hener P Michea P Karasuyama H Chan S Soumelis V Skin thymic stromal lymphopoietin initiates Th2 responses through an orchestrated immune cascade. Nat Commun. (2013) 4:2847. 10.1038/ncomms3847 24284909

[21] Byrd AL Belkaid Y Segre JA. The human skin microbiome. Nat Rev Microbiol. (2018) 16(3):143–55. 10.1038/nrmicro.2017.157 29332945

[22] Bjerre RD Holm JB Palleja A Sølberg J Skov L Johansen JD. Skin dysbiosis in the microbiome in atopic dermatitis is site-specific and involves bacteria, fungus and virus. BMC Microbiol. (2021) 21(1):256. 10.1186/s12866-021-02302-2 34551705 PMC8459459

[23] Schneider AM Nelson AM. Skin microbiota: friend or foe in pediatric skin health and skin disease. Pediatr Dermatol. (2019) 36(6):815–22. 10.1111/pde.13955 31588632

[24] Nakatsuji T Gallo RL. The role of the skin microbiome in atopic dermatitis. Ann Allergy Asthma Immunol. (2019) 122(3):263–9. 10.1016/j.anai.2019.08.025 30550810 PMC7147826

[25] Rodríguez-Tamayo EA Jiménez-Quiceno JN. Factors related with colonization by *Staphylococcus aureus*. Iatreia. (2015) 28(1):66–77. 10.17533/udea.iatreia.18007

[26] Tauber M Balica S Hsu CY Jean-Decoster C Lauze C Redoules D *Staphylococcus aureus* density on lesional and nonlesional skin is strongly associated with disease severity in atopic dermatitis. J Allergy Clin Immunol. (2016) 137(4):1272–1274.e3. 10.1016/j.jaci.2015.07.052 26559326

[27] Kim J Kim BE Ahn K Leung DYM. Interactions between atopic dermatitis and *Staphylococcus aureus* infection: clinical implications. Allergy Asthma Immunol Res. (2019) 11(5):593–603. 10.4168/aair.2019.11.5.593 31332972 PMC6658404

[28] Kennedy EA Connolly J Hourihane JO Fallon PG McLean WHI Murray D Skin microbiome before development of atopic dermatitis: early colonization with commensal staphylococci at 2 months is associated with a lower risk of atopic dermatitis at 1 year. J Allergy Clin Immunol. (2017) 139(1):166–72. 10.1016/j.jaci.2016.07.029 27609659 PMC5207796

[29] Nakatsuji T Chen TH Narala S Chun KA Two AM Yun T Antimicrobials from human skin commensal bacteria protect against *Staphylococcus aureus* and are deficient in atopic dermatitis. Sci Transl Med. (2017) 9((378):eaah4680. 10.1126/scitranslmed.aah4680 28228596 PMC5600545

[30] Byrd AL Deming C Cassidy SKB Harrison OJ Ng WI Conlan S *Staphylococcus aureus* and Staphylococcus epidermidis strain diversity underlying pediatric atopic dermatitis. Sci Transl Med. (2017) 9(397):eaal4651. 10.1126/scitranslmed.aal4651 28679656 PMC5706545

[31] Anania C Brindisi G Martinelli I Bonucci E D'Orsi M Ialongo S Probiotics function in preventing atopic dermatitis in children. Int J Mol Sci. (2022) 23(10):5409. 10.3390/ijms23105409 35628229 PMC9141149

[32] Khadka VD Key FM Romo-González C Martínez-Gayosso A Campos-Cabrera BL Gerónimo-Gallegos A The skin microbiome of patients with atopic dermatitis normalizes gradually during treatment. Front Cell Infect Microbiol. (2021) 11:720674. 10.3389/fcimb.2021.720674 34631601 PMC8498027

[33] Ellis SR Nguyen M Vaughn AR Notay M Burney WA Sandhu S The skin and gut microbiome and its role in common dermatologic conditions. Microorganisms. (2019) 7(11):550. 10.3390/microorganisms7110550 31717915 PMC6920876

[34] Saheb Kashaf S Proctor DM Deming C Saary P Hölzer M, NISC Comparative Sequencing Program, et al. Integrating cultivation and metagenomics for a multi-kingdom view of skin microbiome diversity and functions. Nat Microbiol. (2022) 7(1):169–79. 10.1038/s41564-021-01011-w 34952941 PMC8732310

[35] Chaudhary PP Myles IA Zeldin J Dabdoub S Deopujari V Baveja R Shotgun metagenomic sequencing on skin microbiome indicates dysbiosis exists prior to the onset of atopic dermatitis. Allergy. (2023) 78(10):2724–31. 10.1111/all.15806 37422700 PMC10543534

[36] Shi B Bangayan NJ Curd E Taylor PA Gallo RL Leung DYM The skin microbiome is different in pediatric versus adult atopic dermatitis. J Allergy Clin Immunol. (2016) 138:1233–6. 10.1016/j.jaci.2016.04.053 27474122 PMC5235385

[37] Katsarou A Armenaka M. Atopic dermatitis in older patients: particular points. J Eur Acad Dermatol Venereol. (2011) 25(1):12–8. 10.1111/j.1468-3083.2010.03737.x 20569298

[38] Ndhlovu GON Abotsi RE Shittu AO Abdulgader SM Jamrozy D Dupont CL Molecular epidemiology of *Staphylococcus aureus* in African children from rural and urban communities with atopic dermatitis. BMC Infect Dis. (2021) 21(1):348. 10.1186/s12879-021-06044-4 33849482 PMC8045247

[39] Galkin F Mamoshina P Aliper A Putin E Moskalev V Gladyshev VN Human gut microbiome aging clock based on taxonomic profiling and deep learning. iScience. (2020) 23(6):101199. 10.1016/j.isci.2020.101199 32534441 PMC7298543

[40] Lee SY Lee E Park YM Hong SJ. Microbiome in the gut-skin axis in atopic dermatitis. Allergy Asthma Immunol Res. (2018) 10(4):354–62. 10.4168/aair.2018.10.4.354 29949831 PMC6021588

[41] Bhatt M Lal K Silverberg NB. Special considerations in atopic dermatitis in young children. Dermatol Clin. (2024) 42(4):611–7. 10.1016/j.det.2024.05.003 39278714

[42] Jackson MA Verdi S Maxan ME Shin CM Zierer J Bowyer RCE Gut microbiota associations with common diseases and prescription medications in a population-based cohort. Nat Commun. (2018) 9(1):2655. 10.1038/s41467-018-05184-7 29985401 PMC6037668

[43] Mahmud MR Akter S Tamanna SK Mazumder L Esti IZ Banerjee S Impact of gut microbiome on skin health: gut-skin axis observed through the lenses of therapeutics and skin diseases. Gut Microbes. (2022) 14(1):2096995. 10.1080/19490976.2022.2096995 35866234 PMC9311318

[44] Huang AH Roh YS Sutaria N Choi J Williams KA Canner JK Real-world comorbidities of atopic dermatitis in the pediatric ambulatory population in the United States. J Am Acad Dermatol. (2021) 85(4):893–900. 10.1016/j.jaad.2021.03.016 33689777

[45] Hong PY Lee BW Aw M Shek LP Yap GC Chua KY Comparative analysis of fecal microbiota in infants with and without eczema. PLoS One. (2010) 5(4):e9964. 10.1371/journal.pone.0009964 20376357 PMC2848600

[46] Gore C Munro K Lay C Bibiloni R Morris J Woodcock A Bifidobacterium pseudocatenulatum is associated with atopic eczema: a nested case-control study investigating the fecal microbiota of infants. J Allergy Clin Immunol. (2008) 121(1):135–40. 10.1016/j.jaci.2007.07.061 17900682

[47] West CE Rydén P Lundin D Engstrand L Tulic MK Prescott SL. Gut microbiome and innate immune response patterns in IgE-associated eczema. Clin Exp Allergy. (2015) 45(9):1419–29. 10.1111/cea.12566 25944283

[48] Penders J Gerhold K Stobberingh EE Thijs C Zimmermann K Lau S Establishment of the intestinal microbiota and its role for atopic dermatitis in early childhood. J Allergy Clin Immunol. (2013) 132(3):601–607.e8. 10.1016/j.jaci.2013.05.043 23900058

[49] Song H Yoo Y Hwang J Na YC Kim HS. Faecalibacterium prausnitzii subspecies-level dysbiosis in the human gut microbiome underlying atopic dermatitis. J Allergy Clin Immunol. (2016) 137(3):852–60. 10.1016/j.jaci.2015.08.021 26431583

[50] Wang Y Hou J Tsui JC Wang L Zhou J Chan UK Unique gut microbiome signatures among adult patients with moderate to severe atopic dermatitis in Southern Chinese. Int J Mol Sci. (2023) 24(16):12856. 10.3390/ijms241612856 37629036 PMC10454836

[51] Díez-Madueño K de la Cueva Dobao P Torres-Rojas I Fernández-Gosende M Hidalgo-Cantabrana C Coto-Segura P Gut dysbiosis and adult atopic dermatitis: a systematic review. J Clin Med. (2024) 14(1):19. 10.3390/jcm14010019 39797102 PMC11721037

[52] Ren Z Silverberg JI. Association of atopic dermatitis with bacterial, fungal, viral, and sexually transmitted skin infections. Dermatitis. (2020) 31(2):157–64. 10.1097/DER.0000000000000526 32049716

[53] Silverberg JI Vakharia PP Chopra R Sacotte R Patel N Immaneni S Phenotypical differences of childhood- and adult-onset atopic dermatitis. J Allergy Clin Immunol Pract. (2018) 6(4):1306–12. 10.1016/j.jaip.2017.10.005 29133223 PMC5945342

[54] Ramírez-Marín HA Silverberg JI. Differences between pediatric and adult atopic dermatitis. Pediatr Dermatol. (2022) 39(3):345–53. 10.1111/pde.14971 35297082

[55] Chatrath S Silverberg JI. Phenotypic differences of atopic dermatitis stratified by age. JAAD Int. (2022) 11:1–7. 10.1016/j.jdin.2022.08.026 36818679 PMC9932465

[56] Silverberg JI Silverberg NB. Childhood atopic dermatitis and warts are associated with increased risk of infection: a US population-based study. J Allergy Clin Immunol. (2014) 133(4):1041–7. 10.1016/j.jaci.2013.08.012 24094542

[57] von Kobyletzki L Henrohn D Ballardini N Neary MP Ortsäter G Rieem Dun A Comorbidities in childhood atopic dermatitis: a population-based study. J Eur Acad Dermatol Venereol. (2024) 38(2):354–64. 10.1111/jdv.19569 37824103

[58] Edslev SM Agner T Andersen PS. Skin microbiome in atopic dermatitis. Acta Derm Venereol. (2020) 100(12):adv00164. 10.2340/00015555-3514 32419029 PMC9189751

[59] Hartman-Adams H Banvard C Juckett G. Impetigo: diagnosis and treatment. Am Fam Physician. (2014) 90(4):229–35. 25250996

[60] Mannschreck D Feig J Selph J Cohen B. Disseminated bullous impetigo and atopic dermatitis: case series and literature review. Pediatr Dermatol. (2020) 37(1):103–8. 10.1111/pde.14032 31755570

[61] Wang V Keefer M Ong PY. Antibiotic choice and methicillin-resistant *Staphylococcus aureus* rate in children hospitalized for atopic dermatitis. Ann Allergy Asthma Immunol. (2019) 122(3):314–7. 10.1016/j.anai.2018.12.001 30529713

[62] Totte JE van-der-Feltz WT Hennekam M Belkum A van-Zuuren EJ Pasmans SG. Prevalence and odds of Staphylococcus aureus carriage in atopic dermatitis: a systematic review and meta-analysis. Br J Dermatol. (2016) 175(4):687–95. 10.1111/bjd.14566 26994362

[63] Alexander H Paller AS Traidl-Hoffmann C Beck LA De Benedetto A Dhar S The role of bacterial skin infections in atopic dermatitis: expert statement and review from the international eczema council skin infection group. Br J Dermatol. (2020) 182(6):1331–42. 10.1111/bjd.18643 31677162 PMC7317931

[64] Matsui K Nishikawa A Suto H Tsuboi R Ogawa H. Comparative study of *Staphylococcus aureus* isolated from lesional and non-lesional skin of atopic dermatitis patients. Microbiol Immunol. (2000) 44(11):945–7. 10.1111/j.1348-0421.2000.tb02587.x 11145276

[65] Buda A Międzobrodzki J. The role of *Staphylococcus aureus* in secondary infections in patients with atopic dermatitis (AD). Pol J Microbiol. (2016) 65(3):253–9. 10.5604/17331331.1215600 29334062

[66] Juhn YJ Frey D Li X Jacobson R. Streptococcus pyogenes upper respiratory infection and atopic conditions other than asthma: a retrospective cohort study. Prim Care Respir J. (2012) 21(2):153–8. 10.4104/pcrj.2011.00110 22270478 PMC6547913

[67] Shayegan LH Richards LE Morel KD Levin LE. Punched-out erosions with scalloped borders: group A streptococcal pustulosis. Pediatr Dermatol. (2019) 36(6):995–6. 10.1111/pde.13956 31410890

[68] Gao PS Rafaels NM Hand T Murray T Boguniewicz M Hata T Filaggrin mutations that confer risk of atopic dermatitis confer greater risk for eczema herpeticum. J Allergy Clin Immunol. (2009) 124(3):507–13. 513.e1–7. 10.1016/j.jaci.2009.07.034 19733298 PMC5103856

[69] Desai AD Tefft K Heath CR Lipner SR. Bacterial infections are common among eczema herpeticum inpatients in a cross-sectional analysis of the 2016 Kids’ inpatient database. Pediatr Dermatol. (2023) 40(5):873–6. 10.1111/pde.15395 37437893

[70] Beck LA Boguniewicz M Hata T Schneider LC Hanifin J Gallo R Phenotype of atopic dermatitis subjects with a history of eczema herpeticum. J Allergy Clin Immunol. (2009) 124(2):260–9. 269.e1–7. 10.1016/j.jaci.2009.05.020 19541356 PMC3056058

[71] Bin L Kim BE Brauweiler A Goleva E Streib J Ji Y *Staphylococcus aureus α*-toxin modulates skin host response to viral infection. J Allergy Clin Immunol. (2012) 130(3):683–691.e2. 10.1016/j.jaci.2012.06.019 22840852 PMC3594992

[72] Wang V Boguniewicz J Boguniewicz M Ong PY. The infectious complications of atopic dermatitis. Ann Allergy Asthma Immunol. (2021) 126(1):3–12. 10.1016/j.anai.2020.08.002 32771354 PMC7411503

[73] Mathes EF Oza V Frieden IJ Cordoro KM Yagi S Howard R “Eczema coxsackium” and unusual cutaneous findings in an enterovirus outbreak. Pediatrics. (2013) 132(1):e149–57. 10.1542/peds.2012-3175 23776120 PMC4074616

[74] Olsen JR Piguet V Gallacher J Francis NA. Molluscum contagiosum and associations with atopic eczema in children: a retrospective longitudinal study in primary care. Br J Gen Pract. (2016) 66(642):e53–58. 10.3399/bjgp15×688093 26639950 10.3399/bjgp15X688093 PMC4684036

[75] Zhang LQ Zhang YT Tan C. Molluscum contagiosum with halo dermatitis. J Allergy Clin Immunol Pract. (2021) 9(10):3805–6. 10.1016/j.jaip.2021.06.038 34305042

[76] Silverberg NB. Molluscum contagiosum virus infection can trigger atopic dermatitis disease onset or flare. Cutis. (2018) 102(3):191–4. 30372710

[77] Fan R Leasure AC Damsky W Cohen JM. Association between atopic dermatitis and COVID-19 infection: a case-control study in the all of US research program. JAAD Int. (2022) 6:77–81. 10.1016/j.jdin.2021.12.007 34977817 PMC8712258

[78] van Buchem-Post NF Ouwerkerk W Stalman EW van Dam KPJ Wieske L Bekkenk MW Impact of COVID-19 disease and vaccination on dermatological immune-mediated inflammatory diseases atopic dermatitis, psoriasis, and vitiligo: a Target2B! substudy. J Dermatol. (2025):1–10. 10.1111/1346-8138.17664 39950702 PMC11975183

[79] Patrick MT Zhang H Wasikowski R Prens EP Weidinger S Gudjonsson JE Associations between COVID-19 and skin conditions identified through epidemiology and genomic studies. J. Allergy Clin. Immunol. (2021) 147:857–869.e7. 10.1016/j.jaci.2021.01.006 33485957 PMC7825803

[80] Schmitt J Ehm F Vivirito A Wende D Batram M Loser F Large cohort study shows increased risk of developing atopic dermatitis after COVID-19 disease. Allergy. (2024) 79(1):232–4. 10.1111/all.15827 37469301

[81] Tang Z Chen Y Ouyang Y Peng Y Man X. COVID-19 related epigenetic changes and atopic dermatitis: an exploratory analysis. World Allergy Organ J. (2025) 18(1):101022. 10.1016/j.waojou.2024.101022 39867872 PMC11758953

[82] Sugita T Suto H Unno T Tsuboi R Ogawa H Shinoda T Molecular analysis of malassezia microflora on the skin of atopic dermatitis patients and healthy subjects. J Clin Microbiol. (2001) 39(10):3486–90. 10.1128/JCM.39.10.3486-3490.2001 11574560 PMC88376

[83] Hiragun M Hiragun T Ishii K Suzuki H Tanaka A Yanase Y Elevated serum IgE against MGL_1304 in patients with atopic dermatitis and cholinergic urticaria. Allergol Int. (2014) 63(1):83–93. 10.2332/allergolint.13-OA-0611 24457815

[84] Chong AC Navarro-Triviño FJ Su M Park CO. Fungal head and neck dermatitis: current understanding and management. Clin Rev Allergy Immunol. (2024) 66(3):363–75. 10.1007/s12016-024-09000-7 39031274 PMC11422441

[85] Halkjaer LB Loland L Buchvald FF Agner T Skov L Strand M Development of atopic dermatitis during the first 3 years of life: the Copenhagen prospective study on asthma in childhood cohort study in high-risk children. Arch Dermatol. (2006) 142(5):561–6. 10.1001/archderm.142.5.561 16702493

[86] Ishibashi Y Sugita T Nishikawa A. Cytokine secretion profile of human keratinocytes exposed to malassezia yeasts. FEMS Immunol Med Microbiol. (2006) 48(3):400–9. 10.1111/j.1574-695X.2006.00163.x 17069617

[87] Brough HA Lanser BJ Sindher SB Teng JMC Leung DYM Venter C Early intervention and prevention of allergic diseases. Allergy. (2022) 77(2):416–41. 10.1111/all.15006 34255344

[88] Kim KH. Clinical pearls from atopic dermatitis and its infectious complications. Br J Dermatol. (2014) 170(Suppl 1):25–30. 10.1111/bjd.12919 24720465

[89] Choi Y Park KY Han HS Lee MK Seo SJ. Comparative analysis of cutaneous fungi in atopic dermatitis patients and healthy individuals. Ann Dermatol. (2022) 34(2):118–24. 10.5021/ad.2022.34.2.118 35450318 PMC8989898

[90] Lewis-Jones S Gilmour E Cork MJ Clarck C Cox H Lawton S Atopic Eczema in Under 12s: Diagnosis and Management. London: National Institute for Health and Care Excellence (NICE) (2023). NICE Clinical Guidelines, No. 57. 34101396

[91] Eichenfield LF Stripling S Fung S Cha A O'Brien A Schachner LA. Recent developments and advances in atopic dermatitis: a focus on epidemiology, pathophysiology, and treatment in the pediatric setting. Paediatr Drugs. (2022) 24(4):293–305. 10.1007/s40272-022-00499-x 35698002 PMC9191759

[92] Bath-Hextall FJ Birnie AJ Ravenscroft JC Williams HC. Interventions to reduce *Staphylococcus aureus* in the management of atopic eczema: an updated cochrane review. Br J Dermatol. (2010) 163(1):12–26. 10.1111/j.1365-2133.2010.09743.x 20222931

[93] Hogan PG Mork RL Thompson RM Muenks CE Boyle MG Sullivan ML Environmental methicillin-resistant *Staphylococcus aureus* contamination, persistent colonization, and subsequent skin and soft tissue infection. JAMA Pediatr. (2020) 174(6):552–62. 10.1001/jamapediatrics.2020.0132 32227144 PMC7105954

[94] Elizalde-Jiménez IG Ruiz-Hernández FG Carmona-Cruz SA Pastrana-Arellano E Aquino-Andrade A Romo-González C Global antimicrobial susceptibility patterns of *Staphylococcus aureus* in atopic dermatitis: a systematic review and meta-analysis. JAMA Dermatol. (2024) 160(11):1171–81. 10.1001/jamadermatol.2024.3360 39320869 PMC11425196

[95] Cavalcante FS Saintive S Carvalho Ferreira D Rocha Silva AB Guimarães LC Braga BS Methicillin-resistant *Staphylococcus aureus* from infected skin lesions present several virulence genes and are associated with the CC30 in Brazilian children with atopic dermatitis. Virulence. (2021) 12(1):260–9. 10.1080/21505594.2020.1869484 33356835 PMC7808431

[96] Lepoutre A Doloy A Bidet P Leblond A Perrocheau A Bingen E Microbiologists of the epibac network. Epidemiology of invasive Streptococcus pyogenes infections in France in 2007. J Clin Microbiol. (2011) 49(12):4094–100. 10.1128/JCM.00070-11 21976764 PMC3232948

[97] Kimberlin D Brady M Jackson M Long S. Red Book: 2018 Report of the Committee of Infectious Diseases. Itasca, IL: American Academy of Pediatrics (2018).

[98] Hemani SA Edmond MB Jaggi P Cooley A. Frequency and clinical features associated with eczema herpeticum in hospitalized children with presumed atopic dermatitis skin infection. Pediatr Infect Dis J. (2020) 39(4):263–6. 10.1097/INF.0000000000002542 31764378

[99] Can B Topaloğlu F Kavala M Turkoglu Z Zindancı I Sudogan S. Treatment of pediatric molluscum contagiosum with 10% potassium hydroxide solution. J Dermatolog Treat. (2014) 25(3):246–8. 10.3109/09546634.2012.697988 22639976

[100] Gürel Dİ Soyer Ö Şahiner ÜM. Systemic treatments in atopic dermatitis in children. Turk J Pediatr. (2023) 65(6):887–905. 10.24953/turkjped.2023.203 38204304

[101] Czarnowicki T Malajian D Khattri S Correa-da-Rosa J Dutt R Finney R Petrolatum: barrier repair and antimicrobial responses underlying this “inert” moisturizer. J Allergy Clin Immunol. (2016) 137(4):1091–1102.e7. 10.1016/j.jaci.2015.08.013 26431582

[102] Huang JT Rademaker A Paller AS. Dilute bleach baths for *Staphylococcus aureus* colonization in atopic dermatitis to decrease disease severity. Arch Dermatol. (2011) 147(2):246–7. 10.1001/archdermatol.2010.434 21339459

[103] Bakaa L Pernica JM Couban RJ Tackett KJ Burkhart CN Leins L Bleach baths for atopic dermatitis: a systematic review and meta-analysis including unpublished data, Bayesian interpretation, and GRADE. Ann Allergy Asthma Immunol. (2022) 128(6):660–668.e9. 10.1016/j.anai.2022.03.024 35367346

[104] Moreau M Seité S Aguilar L Da Cruz O Puech J Frieling J Topical *S. aureus*—targeting endolysin significantly improves symptoms and QoL in individuals with atopic dermatitis. J Drugs Dermatol. (2021) 20(12):1323–8. 10.36849/jdd.6363 34898160

[105] Joshi AA Vocanson M Nicolas JF Wolf P Patra V. Microbial derived antimicrobial peptides as potential therapeutics in atopic dermatitis. Front Immunol. (2023) 14:1125635. 10.3389/fimmu.2023.1125635 36761743 PMC9907850

[106] Weiss A Delavenne E Matias C Lagler H Simon D Li P Topical niclosamide (ATx201) reduces *Staphylococcus aureus* colonization and increases shannon diversity of the skin microbiome in atopic dermatitis patients in a randomized, double-blind, placebo-controlled phase 2 trial. Clin Transl Med. (2022) 12(5):e790. 10.1002/ctm2.790 35522900 PMC9076020

[107] Woo TE Sibley CD. The emerging utility of the cutaneous microbiome in the treatment of acne and atopic dermatitis. J Am Acad Dermatol. (2020) 82(1):222–8. 10.1016/j.jaad.2019.08.078 31499149

[108] Belete MA Tadesse S Tilahun M Alemayehu E Saravanan M. Phage endolysins as new therapeutic options for multidrug resistant *Staphylococcus aureus*: an emerging antibiotic-free way to combat drug resistant infections. Front Cell Infect Microbiol. (2024) 14:1397935. 10.3389/fcimb.2024.1397935 38953006 PMC11215010

[109] Hung SH Lin YT Chu CY Lee CC Liang TC Yang YH Staphylococcus colonization in atopic dermatitis treated with fluticasone or tacrolimus with or without antibiotics. Ann Allergy Asthma Immunol. (2007) 98(1):51–6. 10.1016/S1081-1206(10)60859-9 17225720

[110] Callewaert C Nakatsuji T Knight R Kosciolek T Vrbanac A Kotol P IL-4R*α* blockade by dupilumab decreases *Staphylococcus aureus* colonization and increases microbial diversity in atopic dermatitis. J Invest Dermatol. (2020) 140(1):191–202.e7. 10.1016/j.jid.2019.05.024 31252032 PMC7163930

[111] Hoy SM. Ruxolitinib cream 1.5%: a review in mild to moderate atopic dermatitis. Am J Clin Dermatol. (2023) 24(1):143–51. Erratum in: Am J Clin Dermatol. 2023;24(3):495. doi: 10.1007/s40257-023-00769-5. 10.1007/s40257-022-00748-2 36538235 PMC10036407

[112] Torrelo A Rewerska B Galimberti M Paller A Yang CY Prakash A Efficacy and safety of baricitinib in combination with topical corticosteroids in paediatric patients with moderate-to-severe atopic dermatitis with an inadequate response to topical corticosteroids: results from a phase III, randomized, double-blind, placebo-controlled study (BREEZE-AD PEDS). Br J Dermatol. (2023) 189(1):23–32. 10.1093/bjd/Ljad096 36999560

[113] Navarrete-Rodríguez EM Larenas-Linnemann D de la Cruz H V Luna-Pech JA Guevara Sanginés E. Oral janus kinase inhibitors in pediatric atopic dermatitis. Curr Allergy Asthma Rep. (2024) 24(9):485–96. 10.1007/s11882-024-01167-5 39105881

[114] Nakagawa H Igarashi A Saeki H Kabashima K Tamaki T Kaino H Safety, efficacy, and pharmacokinetics of delgocitinib ointment in infants with atopic dermatitis: a phase 3, open-label, and long-term study. Allergol Int. (2024) 73(1):137–42. 10.1016/j.alit.2023.04.003 37100717

[115] Molla A. A comprehensive review of phototherapy in atopic dermatitis: mechanisms, modalities, and clinical efficacy. Cureus. (2024) 16(3):e56890. 10.7759/cureus.56890 38665759 PMC11043791

[116] Legat FJ. Itch in atopic dermatitis—what is new? Front Med (Lausanne). (2021) 8:644760. 10.3389/fmed.2021.644760 34026782 PMC8137993

